# The Molecular Genetics and Genomics of Plant–Pathogen Interactions

**DOI:** 10.3390/ijms25073970

**Published:** 2024-04-03

**Authors:** Vijai Bhadauria, Wensheng Zhao

**Affiliations:** 1 Department of Plant Pathology, College of Plant Protection, China Agricultural University, Beijing 100193, China; 2 Ministry of Agriculture and Rural Affairs—Key Laboratory for Crop Pest Monitoring and Green Control, China Agricultural University, Beijing 100193, China

Plants have evolved an intricate immune system to protect themselves from potential pathogens. They rely on their innate immunity, composed of cell surface and intracellular receptors, to detect the conserved molecular signatures (pathogen-associated molecular patterns [PAMPs]) of potential pathogens. This so-called PAMP-triggered immunity mounts an effective immune response against a vast majority of potential pathogens. However, pathogens can infect plants by subduing the immune response using their effectors, i.e., small proteins secreted into the host cell cytoplasm. In response, plants have evolved intracellular receptors called NLR receptors to detect these effectors and activate effector-triggered immunity [1,2]. This interaction between effectors and the plant immune network determines the outcome of plant–pathogen interactions regarding susceptibility or resistance. The plant immune system is a dynamic system that is constantly evolving to protect plants from potential environmental threats.

This Special Issue delves into the molecular genetics and genomics of these complex plant–pathogen interactions and has garnered a significant number of submissions, encompassing scope-specific topics such as pathogenomics, genomics of emerging pathogens of crops, effector biology, virulence/pathogenicity, plant disease resistance and genetic sources of disease resistance, molecular mechanisms underlying plant–pathogen interactions, technologies for dissecting plant disease resistance and pathogen virulence/pathogenicity, the omics (genomics, transcriptomics and proteomics) of plant–pathogen interactions and genetic dissection of crop resistance. Two of the nine articles published are centered on viral pathogens (tomato leaf curl New Delhi virus-ES and pecan mosaic virus); three focus on bacterial pathogens (*Klebsiella pneumoniae*, *Xanthomonas citri* pv. *Citri*, *Candidatus* Liberibacter asiaticus and *Erwinia-Pantoea* clade bacteria); and three discuss fungal pathogens (*Colletotrichum* spp., *Puccinia striiformis* f. sp. *tritici* and *Botrytis cinerea*) and their interactions with hosts. The remaining article describes an enabling technology for high-throughput gene function assays in plants.

Vo and colleagues investigated the infectivity of two clones of the Tomato leaf curl New Delhi virus (ToLCNDV) in cucumbers, which resulted in opposite symptom appearance. They compared the transcriptomes of ToLCNDV-infected and mock-inoculated cucumber plants to identify disease-associated genes that could contribute to symptom development. Differentially expressed genes were higher in ToLCNDV-India-infected plants than in the ToLCNDV-ES-infected samples. The researchers found that flavonoid pathway-related genes were induced in ToLCNDV-ES-infected cucumber plants, but some were repressed in ToLCNDV-India-infected cucumber plants, indicating their role in resistance during ToLCNDV infections. This research offers initial data from RNA-Seq that can be used to explore various ToLCNDV infections in more detail.

The pecan leaf-variegated plant infected with the novel badnavirus pecan mosaic virus (PMV) is an excellent model plant for studying the mechanisms that lead to the retention of greenery or chlorosis of virus-infected leaves. Zhang et al. found that PMV infection induces both PAMP-triggered immunity and effector-triggered immunity in pecan leaves. The fatty acid-derived signaling pathway is critical in response to PMV infection, as the virus suppresses key genes implicated in fatty acid biosynthesis. Additionally, the salicylic acid biosynthesis pathway is blocked, while cytokinin biosynthesis and signaling are remarkably strengthened in PMV-infected green leaves. The enhanced cytokinin accumulation in green leaves activated plant immune responses and induced partial salicylic acid biosynthesis, leading to comparatively higher systemic acquired resistance compared to that of yellow leaves. These findings provide new insights into the molecular mechanisms underlying antiviral immunity in pecans and suggest potential targets for developing effective strategies to manage PMV infections in pecan plants.

*K. pneumoniae* is a pathogen that can infect both humans and animals, and can be transmitted through food [3,4]. Huang and colleagues discovered that this pathogen can also infect plants, such as maize, banana and pomegranate. The study uncovered a specific strain of *K. pneumoniae* isolated from maize found to have various virulence and resistance genes contributing to its ability to disrupt plant defense mechanisms. Of particular interest is the *rcsB* gene, which is responsible for disease in both plants and animals. This finding shed light on the molecular mechanisms involved in plant–pathogen interactions, highlighting the need for further research on the functions of these genes.

The ETHYLENE INSENSITIVE3-LIKE (EIL) family of transcription factors plays a critical role in various plant physiological and biochemical processes [5]. Su et al. analyzed ten EIL transcription factors in sweet orange and found that they are involved in plant development and responses to biotic and abiotic stress. The EIL transcription factor-coding genes were expressed in different organs of sweet orange and responded to high and low temperatures, NaCl treatment and ethylene-dependent transcription. Additionally, some genes showed pronounced upregulation in response to citrus canker and Citrus Huanglongbing. These findings offer insights into the potential roles of the EIL family in disease resistance.

Tomar and colleagues investigated YopJ effectors from phytopathogens [6,7,8], which revealed the ways in which these proteins modify and suppress their host defense targets. However, the related group of effectors (e.g., Eop1s) has been relatively neglected. This is surprising given their widespread presence in the *Erwinia*-*Pantoea* clade, which contains phytopathogens, non-pathogens and potential biocontrol agents. Our team conducted a comprehensive comparative analysis of Eop1 group effectors using AlphaFold modeling in planta transient expressions and targeted mutational analyses. The results shed light on several new findings, including putative binding sites for inositol hexakisphosphate and acetyl coenzyme A, and newly postulated target-binding domains. Additionally, the study raises questions about whether these effectors function through a catalytic triad mechanism and suggests that some Eop1s may use a catalytic dyad acetylation mechanism promoted by the electronegative environment around the active site. This research provides insights into the roles of these understudied effectors and has important implications for agroecological and pathological adaptations.

Bhadauria et al. reviewed the role of minichromosomes in fungal virulence, investigating three *Colletotrichum* spp. (*C. graminicola*, *C. higginsianum* and *C. lentis*). Anthracnose disease caused by *Colletotrichum* spp. is a significant threat to many economically important crops. These fungi have genomes distributed among ten core chromosomes and two to three minichromosomes, but the involvement of the latter in fungal growth, development and virulence is still uncertain. Recent studies have outlined the unique characteristics of the minichromosomes of three hemibiotrophic *Colletotrichum* pathogens. These pathogens harbor one conditionally dispensable minichromosome, which is indispensable for fungal virulence. The minichromosomes are highly compartmentalized into AT-rich and GC-rich blocks, which may help protect them from erosion of already-scarce genes, thereby helping *Colletotrichum* pathogens maintain adaptability to hosts [9,10,11]. These findings may pave the way for new strategies to control anthracnose disease in crops.

Wheat production is significantly limited by stripe rust disease caused by the biotrophic fungus *P. striiformis* f. sp. *tritici* (*Pst*) [12]. However, to combat *Pst*, plants have evolved complex defense mechanisms, such as programmed cell death and hypersensitive response (HR). Transcription factors (TFs) are known to play critical roles in plant defense response [13,14], and Myeloblastosis (MYB) TFs are among the largest TFs families involved in this mechanism [15]. Hawku and colleagues identified a new R2R3 MYB TF from wheat, called TaMYB391, and characterized its functional role during wheat–Pst interaction. The study concluded that *TaMYB391* is upregulated following *Pst* infection and the exogenous application of salicylic acid (SA) and abscisic acid. Moreover, TaMYB391 functions as a positive regulator of HR-associated cell death and confers the resistance of wheat to the stripe rust fungus by regulating certain pathogenesis-related genes, possibly through SA signaling pathways. This study represents a significant advancement toward deciphering the mechanisms of MYB TFs during wheat–*Pst* interactions and provides promising insights for developing new strategies to enhance wheat resistance to the stripe rust fungus.

*B*. *cinerea*, the pathogen responsible for the gray mold disease, is a major threat to cherry tomato crops, causing significant economic loss. Fludioxonil is a widely used fungicide that has been effective in controlling this disease. However, the emergence of resistant strains has made control more difficult. Liu et al. used RNA sequencing to identify genes that play a role in fludioxonil resistance in *B. cinerea*. They found several genes, including transporters and HOG pathway homologs, differentially expressed in fludioxonil-resistant strains. These findings provide valuable insights into the molecular mechanisms of resistance and can be used to develop more effective strategies to control this devastating disease.

*Agrobacterium*-mediated transient expression (AMTE) is a powerful tool for high-throughput gene function assays in plants [16]. However, its application in monocots has been limited due to low expression efficiency. Xu and colleagues optimized the AMTE protocol for barley, wheat and rice plants by investigating the factors affecting the efficiency of gene expression. They found that the vector pCBEP produced the highest expression levels, and concurrent treatments with high humidity and darkness significantly increased efficiency. The optimized AMTE approach was then used to construct a full-length cDNA library of genes upregulated during the rice blast fungus infection and identify 15 candidate genes promoting the blast disease in barley plants. Four of these genes encoded chloroplast-related proteins, and their constitutive overexpression conferred enhanced disease susceptibility to *C. higginsianum* in Arabidopsis. These findings demonstrate the power of the optimized AMTE approach to monocots as an effective tool for facilitating the functional assays of genes mediating complex plant–microbe interactions.

Overall, the molecular genetics and genomics of plant–pathogen interactions are extensively researched in this Special Issue, with studies on viral, bacterial and fungal pathogens shedding light on the mechanisms underlying plant–pathogen interactions and disease resistance.
